# Impact and effectiveness of meningococcal vaccines: a review

**DOI:** 10.26633/RPSP.2017.158

**Published:** 2017-12-20

**Authors:** Lucia Helena De Oliveira, Barbara Jauregui, Ana Flavia Carvalho, Norberto Giglio

**Affiliations:** 1 Comprehensive Family Immunization Unit Pan American Health Organization, Regional Office of the World Health Organization Washington, DC United States of America Comprehensive Family Immunization Unit, Pan American Health Organization, Regional Office of the World Health Organization, Washington, DC, United States of America.; 2 Albert B. Sabin Vaccine Institute Albert B. Sabin Vaccine Institute Washington, DC United States Albert B. Sabin Vaccine Institute, Washington, DC, United States.; 3 Hospital Gutierrez Hospital Gutierrez Buenos Aires Argentina Hospital Gutierrez, Buenos Aires, Argentina.

**Keywords:** Meningococcal vaccines, immunization programs, decision making, prevention & control, review, Latin America., Vacunas meningococicas, programas de inmunización, toma de decisiones, prevención & control, revision, América Latina, Vacinas meningocócicas, programas de imunização, tomada de decisões, prevenção & controle, revisão, América Latina

## Abstract

**Objectives.:**

*To summarize and critically evaluate the evidence on the impact and effectiveness of meningococcal vaccination programs around the world in order to inform decisionmaking in Latin America and the Caribbean*.

**Methods.:**

*A review of the literature was conducted following several components of the Preferred Reporting Items for Systematic Reviews and Meta-Analyses guidelines. PubMed Central® was searched for papers published in any language from January 1999 – March 2017*.

**Results.:**

*In all, 32 studies were included, most of which evaluated the meningococcal C conjugate vaccine. Fourteen studies measured effectiveness and 30 measured impact. The effectiveness of polysaccharide vaccines was 65% – 83.7% (different age groups), while the effectiveness of the conjugate vaccines was 66% – 100%. Incidence decline of laboratory-confirmed meningococcal disease for the conjugate vaccine ranged from 77% – 100% among different ages groups. The only study that evaluated the protein subunit vaccine reported a vaccine effectiveness of 82.9%*.

**Conclusions.:**

*The studies reviewed show impact and effectiveness of both polysaccharide vaccines and conjugate vaccines on vaccine-serogroup meningococcal disease. The conjugate vaccines, however, show higher impact and effectiveness with longer-lasting protection over the polysaccharide vaccines. Given the variance in potential use of a meningococcal vaccine, epidemiological surveillance systems should be strengthened to inform national decisions*.

## RESUMEN

**Objetivos.:**

Resumir y evaluar críticamente los datos científicos sobre la repercusión y la eficacia de los programas de vacunación antimeningocócica en todo el mundo para orientar la toma de decisiones en América Latina y el Caribe.

**Métodos.:**

Se realizó un examen de la bibliografía siguiendo varios componentes de las directrices correspondientes a los elementos de notificación preferidos para revisiones sistemáticas y metanálisis. Se hicieron búsquedas en PubMed Central® para encontrar documentos publicados en cualquier idioma desde enero de 1999 hasta marzo del 2017.

**Resultados.:**

En total, se incluyeron 32 estudios, en cuya mayoría se evaluaba la vacuna antimeningocócica conjugada contra el serotipo C. En 14 estudios se midió la efectividad y en 30, el impacto. La efectividad de las vacunas polisacarídeas se encontraba entre 65% y 83,7% (grupos etarios diferentes), mientras que la de las vacunas conjugadas, entre 66% y 100%. Gracias a la vacuna conjugada, la disminución de la incidencia de la enfermedad meningocócica confirmada en laboratorio varió entre 77% y 100% en diferentes grupos etarios. En el único estudio en el que se evaluó la vacuna de subunidades proteínicas se notificó que su efectividad era de 82,9%.

**Conclusiones.:**

Los estudios examinados muestran el impacto y la efectividad tanto de las vacunas polisacarídeas como de las vacunas conjugadas en relación con la enfermedad meningocócica causada por los serotipos utilizados en las vacunas. Sin embargo, las vacunas conjugadas demuestran que tienen un mayor impacto y efectividad que las vacunas polisacarídeas para dar una protección más duradera. En vista de la varianza del posible uso de una vacuna antimeningocócica, se deben fortalecer los sistemas de vigilancia epidemiológica para orientar las decisiones nacionales.

## RESUMO

**Objetivos.:**

Sumarizar e analisar de maneira crítica as evidências sobre o impacto e a efetividade dos programas de vacinação contra doença meningocócica em todo o mundo para subsidiar a tomada de decisão na América Latina e no Caribe.

**Métodos.:**

Uma revisão da literatura científica foi realizada de acordo com vários componentes das diretrizes dos Principais itens para relatar revisões sistemáticas e meta-análises (PRISMA). Foi feita uma pesquisa da base de dados PubMed Central® em busca de estudos publicados em qualquer idioma de janeiro de 1999 a março de 2017.

**Resultados.:**

Trinta e dois estudos foram selecionados, sendo que a maioria avaliou a vacina conjugada contra o meningococo C. Quatorze artigos avaliaram a efetividade da vacina e 30, o impacto. A efetividade das vacinas polissacarídicas foi de 65% a 83,7% (em diferentes faixas etárias) e a das vacinas conjugadas foi de 66% a 100%. A redução com o uso da vacina conjugada da incidência de doença meningocócica confirmada por laboratório variou de 77% a 100% em diferentes faixas etárias. O único estudo que avaliou a vacina de subunidade proteica informou uma efetividade de 82,9%.

**Conclusões.:**

Os estudos examinados indicam impacto e efetividade da vacina polissacarídica e da vacina conjugada para doença meningocócica do respectivo sorogrupo vacinal. As vacinas conjugadas demonstram maior impacto e efetividade e proteção mais duradoura em relação às vacinas polissacarídicas. Diante da variação do uso em potencial da vacina meningocócica, os sistemas de vigilância epidemiológica devem ser reforçados visando subsidiar a tomada de decisão das autoridades nacionais.

Meningococcal disease is a global problem that occurs in all countries, but its epidemiology varies substantially by capsular group (1). Virulent strains of *Neisseria Meningitidis* have a polysaccharide capsule, with types A, B, C, W, X, and Y commonly causing invasive infections (2).

The incidence of invasive meningococcal disease (IMD) is highest in children less than 1 year of age and remains relatively high until approximately 5 years. Although the incidence tends to decrease in older children, it usually peaks again during adolescence and young adulthood when individuals are living in close quarters. Incidence again tapers off in older adults. This pattern has been observed at both regional and country levels and with individual serogroups. The morbidity and mortality from IMD is substantial, with a case-fatality rate close to 10% in developed countries (3). Many survivors experience permanent debilitating sequelae, such as hearing loss, neurologic impairments, or limb loss (4).

The development of meningococcal vaccines began in the 1960s with the creation of the polysaccharide vaccines, which are immunogenic in older children and adults, but less so in infants. To cover this younger population, conjugate vaccines were developed in 1999. Polysaccharide vaccines and conjugate vaccines based on the meningococcal capsule are now available against all meningococcal strains related to serogroups A, C, W, and Y. Regarding protection against serogroup B, a broadly protective serogroup B recombinant vaccine has recently been approved in several countries (5, 6).

According to the World Health Organization’s (WHO) Vaccine Preventable Diseases Monitoring System, as of 2016, a total of 39 countries had introduced a meningococcal vaccine in their routine immunization schedules. The first country to introduce the conjugate vaccine was the United Kingdom in 1999. Additionally, 30 countries have introduced a meningococcal vaccine for specific highrisk groups only. Infants and young children less than 2 years of age are targeted in 27 countries, while nine countries target children 2 years of age or older and 11 countries target adolescents. Only five countries have introduced a meningococcal vaccine in a geographically restricted area within the country, while the rest have introduced it nationwide (7, 8).

Some countries in Latin America and the Caribbean have decided to implement a nationwide, routine immunization program using meningococcal vaccines. Brazil was the first to introduce a conjugated meningococcal C vaccine (MenC), while Cuba was the first to introduce an outer-membrane protein B vaccine (9). Afterwards, Puerto Rico and Chile introduced the ACWY tetravalent polysaccharide-protein conjugate vaccines in 2008 and 2012, respectively; Argentina did likewise in 2017 (10, 11).

Monitoring post-licensure impact of meningococcal vaccines is important for documenting appropriate gains in terms of expected morbidity and mortality reduction. This information is scarce and scattered in Latin America and the Caribbean, making it difficult for decisionmakers to base policy on evidence. This review aims to summarize the available evidence on the impact and effectiveness of commercially-available vaccines on meningococcal disease, invasive meningococcal disease, and death due to meningococcus in all age groups, as reported by several countries. The intent is inform decision making among countries of Latin America and the Caribbean that are considering introducing the meningococcal vaccine.

## MATERIALS AND METHODS

### Literature search

A literature search following several components of the Preferred Reporting Items for Systematic Reviews and Meta-Analyses (PRISMA) guidelines^4^ was performed on PubMed Central® (National Library of Medicine, Bethesda, Maryland, United States) to identify all available studies on the effects of meningococcal vaccines on meningococcal disease cases and mortality. The main search terms were: “meningococcal vaccine,” “immunization program,” “mass vaccination,” AND “effectiveness” OR “evaluation” OR “impact” OR “benefits,” “hospitalization,” “notifications,” “decline” OR “reduction.” Terms used for exclusion of papers were: “meningococcal vaccines/economics” OR “immuniza tion/economics” or “cost-benefit analysis” OR “MenAfriVac” OR “disease outbreaks” OR “randomized controlled trial.” The search included all papers published from 1 January 1999 – 15 March 2017, the day on which the search was conducted.

### Inclusion and exclusion criteria

Studies of primary impact and effectiveness of currently available meningococcal vaccines were included, published from January 1999 – March 2017, in any language. The review focused on the meningococcal polysaccharide, polysaccharide-protein conjugate, and protein sub unit vaccines, and considered any serogroup except for serogroup A. The outcomes of interest were clinically-compatible and/or laboratory-confirmed meningococcal cases and deaths due to meningococcal disease.

The following study types were excluded: case series, case reports, and randomized controlled trials; and economic, cost-benefit, and modelling studies. Additionally, studies performed in Africa related to meningococcal A vaccine were excluded for not being relevant to Latin American and the Caribbean, as were those studies specifically targeting patients with sickle cell disease, HIV-infection, or conditions known to affect immune response. Others excluded were: studies that considered only the disease of selected serogroups without denominators, adverse events, or immunogenicity (antibody levels); studies of nasopharyngeal carriage; studies considering only laboratory data; studies with all-cause mortality and hospitalization as primary outcomes; and studies that assessed only nosocomial infections.

### Study selection

Citations were screened by two independent reviewers in a two-step process. First, titles and abstracts were reviewed for duplication and inclusion/exclusion criteria. After eliminating duplicates, the full texts of these papers were obtained to complete the eligibility screening. Second, five citations on which eligibility reviewers disagreed were discussed or assessed by a third reviewer.

By screening the abstracts of the remaining 209 studies, 158 were excluded—30 were not related to meningococcal vaccine; 95 did not measure vaccine impact; 6 were reviews (not primary studies); 3 were reports or press release; 8 were studies of meningococcal A vaccine; 3 were studies of vaccines that are not commercially available; and 13 were modelling/economic evaluations.

**FIGURE 1.**
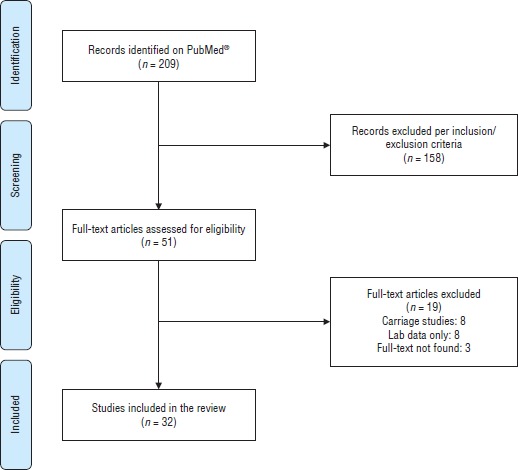
PRISMA Flow Diagram for selection of articles for a review of the literature on the impact and/or effectiveness of meningococcal vaccines, 2016

***Source:*** Prepared by the authors from study data.

The full texts of 51 studies were then evaluated, and another 19 were excluded, resulting in 32 primary studies for this review (12 – 43). Figure 1 shows the PRISMA Flow Diagram of the search strategy and selected articles.

### Data collection and study quality

Data extraction was done independently by three reviewers, using abstraction forms developed specifically for this review. Data extracted included first author, country and state when available, vaccine and serogroup type, study type, data source, study period, vaccine introduction date, vaccination strategy, vaccination schedule used, age groups on which impact was measured, number of subjects on which impact was measured, case definition, effectiveness, or impact as reported, and main conclusion of the study.

The main results included were impact and/or effectiveness assessments, corresponding confidence limits when available, and any information on control and ascertainment of exposure for case-control studies and cohort studies. Additionally, control (as steady practice) and presence of time series before were evaluated for impact studies.

### Data analysis

A descriptive analysis was conducted of each study’s characteristics including design, type, and schedule of meningococcal vaccine introduced, vaccination strategy, data sources, and endpoints considered. For all studies, the main measure of interest was the vaccine effect in reducing the outcome of interest.

## RESULTS

Table 1 summarizes the characteristics of the 32 articles selected. Most of them were spread out evenly through the study period. Of the total, 26 evaluated conjugate vaccines (12 – 37) and 6 evaluated polysaccharide vaccines (38 – 43). By far, the vaccine most commonly evaluated was the meningococcal conjugate C vaccine (Men-C-C), with 25 articles; some studies evaluated multiple vaccines. The studies originated from several countries, with Spain being the most prolific publisher (10 studies), followed by Canada (8 studies). The study periods of the 32 selected articles ranged from 1 – 58 years, with an average of 8.7 years and a median of 5 years. The most common data sources for the studies were surveillance systems (27 studies).

**TABLE 1.** Characteristics of the articles included in a review of the literature on the impact and/or effectiveness of meningococcal vaccines, 2016

Characteristics	*n* (Total 32)	%
Publication year		
1999	1	3
2000	1	3
2001	3	9
2002	0	0
2003	3	9
2004	4	13
2005	2	6
2006	0	0
2007	0	0
2008	1	3
2009	2	6
2010	0	0
2011	2	6
2012	2	6
2013	0	0
2014	5	16
2015	2	6
2016	4	13
Type of vaccine (not mutually exclusive)		
Meningo Conjugate C	25	78
Polysaccharide A+C	4	13
MenACWY-135	2	6
4CMenB	1	3
VA Meningo BC	1	3
Polysaccharide MenC	1	3
Country		
Spain	10	31
Canada	8	25
United Kingdom	4	13
Brazil	3	9
Italy	3	9
Australia	1	3
Cuba	1	3
Ireland	1	3
Scotland	1	3
Study type (not mutually exclusive)		
Impact	30	94
Effectiveness	14	47
Data sources		
Surveillance	27	84
Hospital records	5	16

Regarding the immunization strategy, most countries studied had introduced a meningococcal vaccine to their routine immunization program (25 studies) and 4 had also conducted catch-up for older age groups. Eleven studies evaluated the use of a meningococcal vaccine in a campaign. The vaccination schedule used varied widely. In September 2015, the United Kingdom became the first country to introduce meningococcal vaccine (4CMenB), a protein sub-unit vaccine, using a 2- and 4 months-old schedule with an opportunistic catch-up at 3 and 4 months of age. Among the 26 studies that looked at the conjugate meningococcal vaccines (12 – 37), 23 evaluated routine introductions to national schedules and 6 evaluated campaigns; just a few studies evaluated both. Regarding the routine introduction of conjugate vaccines, the most common schedule used was a single dose at 12 – 15 months of age (11 studies), followed by a three-dose schedule of 2-, 4-, and 6-months of age (6 studies). Four studies also vaccinated adolescents at around 12 years of age. Almost one-half of the conjugate vaccine schedules (12 articles) included a catch-up strategy for older age groups. Regarding the use of conjugate vaccines in campaigns, the targeted ages ranged from 2 months – 20 years of age.

The use of polysaccharides vaccines in the studies identified was predominantly during campaigns (4 of 6 studies), covering a wide range of age groups, usually including infancy – late adolescence. It is noteworthy that these studies were published in the 1990s (38 – 43).

Regarding the estimation of impact or effectiveness of the vaccines, approximately two-thirds of the studies (19 articles) evaluated the impact and/or effectiveness of the vaccine on the entire population, whereas the remaining onethird (13 articles) restricted the measure of effect to a certain age range or specific group. Meningococcal cases with lab confirmation (culture and/or cerebrospinal fluid polymerase chain reaction) were used as the most common endpoint included (25 studies) and secondary data from health information systems, surveillance systems, and other sources were also used. Seven studies evaluated laboratory-confirmed invasive meningococcal disease.

Fourteen studies measured effectiveness by comparing risk or rates in vaccinated and unvaccinated people during the same time period. Thirty studies measured impact by comparing a pre-vaccine introduction period to a post-vaccine introduction period. Regarding the degree of impact of meningococcal vaccines, onethird (11 studies) did not report a measure of change, but rather estimates before and after the introduction. Among the 21 studies that did report a specific measure of impact or change, the actual measure varied, with vaccine effectiveness being the most common measure (14 studies), followed by incidence decline (9 studies), and lastly, relative risk (2 studies). The age range on which the impact was measured also varied within and among studies. A summary of main outcomes and characteristics of the 32 studies included can be found in Tables 2a – d. Among the studies that estimated effectiveness of the polysaccharide vaccines, results ranged from 65% (Canada) – 83.7% (Spain). In the Canada study, vaccine effectiveness was not sustained beyond the second year of the study, showing no effectiveness in years 3 – 5. The only other study that provided an impact estimate for the polysaccharide vaccines was the United Kingdom, reporting a relative risk of 0.94.

With the studies that estimated the effect of the conjugate vaccines, effectiveness estimates ranged from 66% (United Kingdom) – 100% (Spain), and the incidence decline ranged from 77% (Canada) – 100% (Spain). The only study that evaluated the protein sub-unit vaccine reported a vaccine effectiveness of 82.9%.

## DISCUSSION

Overall, the 32 studies included in this review showed a positive impact and effectiveness of both the polysaccharide vaccines and the conjugate vaccines on vaccine-serogroup meningococcal disease and mortality; but the conjugate vaccines show higher impact and effectiveness, plus longer lasting protection over the polysaccharide vaccines. Pelton and colleagues (3) reported similar results, including several studies that showed reductions in vaccine serogroup invasive meningococcal disease incidence after meningococcal polysaccharide vaccines or polysaccharide protein conjugate vaccine introduction, while the overall incidence of other serogroups remained stable, an effect observed in this review’s studies as well. Nonetheless, it is important to consider the advantages of conjugate vaccines, which are well established in regards to immunologic memory and protection in the youngest children (less than 2 years of age) who respond poorly to polysaccharide vaccines. Also, the ability of conjugate vaccines to reduce carriage allows for significant indirect benefits through herd protection, reducing transmission of meningococci (44).

### Study limitations

As mentioned above, this literature review selected effectiveness and impact studies of meningococcal vaccines. Vaccine effectiveness is a measure of protection attributable to a vaccine administered under field conditions to a given population and is measured by observational studies. In turn, impact of a vaccination program is measured by the fraction of prevented disease in a population and is estimated by comparing populations with and without vaccination, usually the same population before and after vaccination. The impact of a vaccination program refers to the program’s overall effect. Different study designs are used for measuring effectiveness and impact, but all the post-licensure study designs present a certain level of bias. For example, in effectiveness studies, the level of natural protection against disease may differ between vaccinees and non-vaccinees, the target group for vaccination may have a higher probability of developing the disease, or the ascertainment of the disease may differ depending on vaccination status. On the other hand, impact studies could enable bias, such as secular trends in baseline transmission or cyclical variations of the target disease, changes in case detection or surveillance methods, or the presence of a concomitant intervention, such as prudent antibiotic use, which has an impact on the target disease as well (45). The majority of the studies included in this review are impact studies using mostly before-and-after design. A study-bystudy assessment of risk bias was not conducted and may represent a limitation to the interpretation of results.

Regarding the reported results, many of the studies did not report a measure of change; among those that did, their measures varied, as did the age range of their study participants. These variations make it difficult to draw comparisons between studies, and even harder to consolidate estimates in an attempt to obtain a “big picture” of the impact of meningococcal vaccines on disease and death due to meningococcus. Additionally, it is worth noting that the countries studied use different vaccine schedules, target a variety of age groups, implement primary vaccination with or without catch-ups, and apply diverse meningococcal disease diagnostic methods, all of which influence results and interpretation.

Of the 32 selected, there was only one study of the impact of the protein subunit vaccine serogroup B 4CMenB (35); this was in part because it was just licensed in 2015. The United Kingdom was the first country to introduce it to the national immunization program (46). During the program’s first 10 months, the two-dose 4CMenB schedule has been highly effective in preventing meningococcal B (MenB) disease in infants.

**TABLE 2a.** 

First Author, year	Country and state	Type of vaccine	Serogroups included in the vaccine	Specific vaccine	Study type	Data source	Study period	Vaccine introduction or campaign DATE
Lawrence, 2016	**Australia**	Conjugate	C	MCCV	Impact	Notification data	2000–2012	2003
Tauil, 2014	**Brazil**, Federal District	Conjugate	C	MCCV	Impact	Surveillance data; mortality database; public health laboratory database	2005–2011	2010
Cardoso, 2012	**Brazil**, Salvador	Conjugate	C	MCCV	Impact and effectiveness	Surveillance	Jan 2000- Dec 2011	1 Feb 2010
Bettinger, 2009	**Canada**	Conjugate	C	MCCV	Impact	Surveillance standardized case report forms from the national Notifiable Diseases Information System (SINAN)]	2002–2006	Staggered implementation starting with campaigns in some provinces in 1999 to 2001, and routine programs starting in 2002 to 2005
De Wals, 2011	**Canada**	Conjugate	C	MCCV	Effectiveness	Laboratory and surveillance data	19 years	2001
Sadarangani, 2014	**Canada**	Conjugate	C	MCCV	Impact	Population based surveillance	1998–2012	2001 – recommendation in Canada
Siu, 2008	**Canada**, British Columbia	Conjugate	C	MCCV	Impact	IMD cases are reported to BCCentre for Disease Control Surveillance Denominator was obtained from an estimation of BC annual population using Population Extrapolation for Organization Planning	2003–2006	2003
Wormsbecke r, 2015	**Canada**, Ontario	Conjugate	C and A C Y W	MCCV and ACWY	Impact	Surveillance data were obtained from the Ontario reportable diseases system, the integrated Public Health Information System (iPHIS), and Public Health Ontario Laboratories (PHOL) for 2000–2013.	2000–2013	2004
Kinlin, 2009	**Canada**, Ontario	Conjugate	C	MCCV	Impact	IMD cases reported to the central public health lab	2000–2006	After 2001
De Wals, 2004	**Canada**, Quebec	Conjugate	C	MCCV	Impact and effectiveness	Population-based observational study of cases of invasive serogroup C meningococcal	1996–2002	2001
O Maoldomhnai gh, 2016	**Ireland**	Conjugate	C	MCCV	Impact	Records from two tertiary pediatric hospitals	2001–2011	1 Oct 2000
De Waure, 2015	**Italy**	Conjugate	C	MCCV	Impact	Two surveillance sources: the Informative System of Infectious Diseases (SIMI) since 1991, and the National Surveillance of Bacterial Meningitis since 1994	1994–2012	2005
Pascucci, 2014	**Italy**, Emilia Romagna	Conjugate	C	MCCV	Impact and effectiveness	Surveillance data	2000–2005	In Emilia-Romagna region: at risk – 2003; in 2006 routine to 12–15 mo and 14–15 yrs. No catch-up
Bechini, 2012	**Italy**, Tuscany	Conjugate	C	MCCV	Impact	Surveillance, The Regional Health Authority supplied all surveillance data on invasive bacterial disease (IBD) of the resident Tuscan population in the same age groups, according to the Italian National Institute for statistics (ISTAT) data	2009-Nov 2011	1 Mar 2005
Mooney, 2004	**Scotland**	Conjugate	C	MCCV	Impact and effectiveness	Statistics Division of the Common Services Agency, National Health Service for ScotlandScottish Meningococcus and Pneumococcus Reference Laboratory,	1994–2003	1999
Cano, 2004	**Spain**	Conjugate	C	MCCV	Impact	Epidemiological surveillance of meningococcal disease in Spain is based on a passive notification system	1999/2000 to 2002/2003	2000
Garrido, 2014	**Spain**	Conjugate	C	MCCV	Impact and effectiveness	Data on cases of meningococcal disease reported in Spain were obtained from the National Notifiable Disease Surveillance System Surveillance	January 2001 to December 2013	1 Dec 2000
Larrauri, 2005	**Spain**	Conjugate	C	MCCV	Impact and effectiveness	Data on cases of meningococcal disease reported in Spain during the study period (1999–2004) were obtained from the Notifiable Disease Surveillance Systempopulation estimates computed at the midpoint of the periods considered and furnished by the National Statistics Institute (Instituto Nacional de Estad’?stica)	1999/2000 to 2001/2003	2000
Salleras 2003 (Impact...)	**Spain**	Conjugate	C	MCCV	Impact	Surveillance data	Jan to Dec 2001	2000
Cruz Rojo, 2005	**Spain**, Andalucia	Conjugate	C	MCCV	Impact	Andalucia meningococcal Survillance and state demographic data	1997–2004	Jul 2000 and expanded in Sep 2001
Salleras, 2003 (Dramatic...)	**Spain**, Catalonia	Conjugate	C	MCCV	Impact and effectiveness	Surveillance data	1999–2000 To 2000–2001 And 2001–2002	2000
Romero, 2011	**Spain**, Galicia	Conjugate	C	MCCV	Impact	Meningitis surveillance system	1990 – 1995	2000: MenC Conjugate
Morales, 2016	**Spain**, Navarra	Conjugate	C	MCCV	Impact and effectiveness	Surveillance data	1995–1999 and 2001–2014	2000
Parikh, 2016	**UK**	Protein Sub Unit Vaccine	B	4CMenB	Impact and Effectiveness	Surveillance	Sept 2015 to June 2016	1 Sep 2015
Martin, 2014	**UK**	Conjugate	C	MCCV	Impact	Routinely collected administrative statistics for hospital care	1963–1998 and 2000–2011	1999
Trotter, 2004	**UK**	Conjugate	C	MCCV	Effectiveness	Cover of Vaccination Evaluated Rapidly (COVER), Child Health Department and National laboratories	2000–2004	1 Nov 1999

**TABLE 2b.** 

First Author, year	Country and state	Specific vaccine	Vaccination STRATEGY (Routine or campaign?)	Vaccination schedule used	Age groups on which impact was measured	Case definition	Effectiveness or impact as reported	Impact estimation	Conclusion of the study (short)
Lawrence, 2016	**Australia**	MCCV	Routine	12 months and catch up for 2 to 19 year-olds	All ages	**Invasive meningococal disease and mortality**	Incidence declined 96%, CI 94–98, and deaths declined from 68 in 2000–2002 to 3 deaths in 2010–2012	incidence decline 96%	Despite published evidence of waning antibody over time, an ongoing single dose of meningococcal C conjugate vaccine in the second year of life following widespread catch-up has resulted in near elimination of serogroup C disease in all age groups without evidence of vaccine failures in the first decade since introduction
Tauil, 2014	**Brazil**, Federal District	MCCV	Routine	3m – 2 years	All ages	Clinical manifestations consistent with MD who met at least one of the following criteria: isolation of Neisseria meningitidis from the cerebrospinal fluid (CSF) or blood; positive immunodiagnostic test for N. meningitidis antigen in the CSF or blood; presence of Gram-negative diplococcus in the CSF; or association or not of purpura fulminans with meningitis	The average annual incidence rate was 2.0/100 000 inhabitants/year (range: 1.3–2.5 per 100, 000) from 2005 to 2009 and 1.8 and 0.8/100 000 inhabitants/year in 2010 and 2011. The average annual incidence rates in children under one year of age were 30.6, 13.3 and 13.1/100 000 inhabitants/year and in childrenwith two years of age were 14.4, 10.8 and 2.7/100 000 inhabitants/year from 2005 to 2009 and in 2010 and 2011, respectively	**Not expressed**	In conclusion, the MCCV strategy implemented in Brazil proved highly effective and had a strong direct impact on the target population. However, incidence and case fatality rates of MD remain high with a wide gap in the risk of dis-ease between poor and affluent areas pointing to the need for periodic adjustments and revaluations of the current strategy
Cardoso, 2012	**Brazil**, Salvador	MCCV	Routine + catch-up	2 and 4 months, with booster in the second year of life, and catch up in 2010 for people aged 10 to 24	All age groups	Lab confirmed meningococcal disease	Among children <5, incidence of serogroup C meningococcal disease fell from 7.5 cases per 100 000 per year during 2008–2009, to 4.0 in 2010 and 2.0 per 100 000 in 2011, and was significantly lower in 2011 than during 2008–2009. Among 10–24 year olds, rates of serogroup C disease were lower in 2011 than in 2010, but were not significantly lower than during 2008–2009 before mass vaccination	**VE 100%**	Low coverage in the population targeted for mass vaccination may have limited impact on ongoing transmission of serogroup C meningococcal disease despite high vaccine effectiveness
Bettinger, 2009	**Canada**	MCCV	first campaigns and afterwards routine.	Varies by province. Quebec and Alberta 2, 4 and 6 or 12 months. Other provinces: 2 and 12 months, or onlly 12 months. Most provinces did catch up to children (varying ages, 9 to 18 year-olds)	All age groups	Lab confirmed invasive meningococcal disease	Rates declined from 0.41 (0.28–0.60) in 2002 to 0.07 (0.02– 0.16) in 2006	**Not expressed**	A substantial decrease in group C incidence occurred in provinces with early MenC immunization programs. Serogroup C incidence remained stable in provinces without MenC programs. We found no evidence of serogroup replacement
De Wals 2011	**Canada**	MCCV	campaign	2 months to 20 years	2m to 20 years	Laboratory confirmed (culture, PCR, CSF, polymerase 2001)	Effectiveness 87.4% (CI 75.4–94.2%)	**VE 87.4%**	Results support current Canadian recommendation to provide booster vaccination for adolescents
Sadarangani 2014	**Canada**	MCCV	Routine	Current schedule: <1 yr (three doses); 12–23 mo and 12–24 yrs (1 dose)	all ages	Admission to hospital and identification of Neisseria meningitidis from a sterile site	Between 2002 and 2005, the incidence of IMD was 0.14 per 100 000 per year for serogroup C and 0.33 per 100 000 per year for all other serogroups combined. Between 2009 and 2012, the incidence decreased by 77% to 0.03 per 100 000 per year for serogroup C (P < .0001), with no significant change in non-C disease	**Incidence decline 77%**	MCCV dramatically reduced the incidence of serogroup C IMD in Canada through both direct and indirect effects. The observation that disease incidence decreased with different schedules suggests that the doses at 12 months (common to all provinces) and adolescence (7 of 8 provinces studied) were critical in achieving disease control
Siu, 2008	**Canada**, British Columbia	MCCV	Routine	Infants and adolescents	All ages	Serogroup-specific invasive meningococcal disease	Average annual incidence of serogroup-C IMD has declined from 0.32/100 000 in 2003 to 0.07/100 000 in 2005 in this age group with a significant downward trend (p=0.05)	**Not expressed**	There is a decreasing trend of pediatric serogroup C invasive meningococcal disease and an increase in median age of serogroup C IMD cases since 2003, most likely explained by protection from immunization
Wormsbecker, 2015	**Canada**, Ontario	MCCV and ACWY135	Routine	1 year-old, 12 year-olds	All ages	Invasive meningococcal disease	There were 161 serogroup C IMD cases and its annual incidence decreased significantly over time(17.2% reduction per year [95% CI: 13.4 to 20.7]). The incidence of serogroup C IMD decreased significantly in children aged 1–17 years in the post-program period, based on age-specific incidence rate ratios (IRRs) and their 95% confidence intervals (CIs). Adolescents 12–16 years had the lowest serogroup C IRR (0.07[95% CI: 0.01 to 0.55]); the rate decreased more than 14-fold between the pre- and post-periods. There were 187 serogroup Y IMD cases and there was a non-significant 1.6% reduction per year [95% CI: -1.9to 5.1]) over the surveillance period. Likewise, there was a non-significant decrease in serogroup Y IMD among persons 12–16 years (MCV4 eligible) in the post-program period	**Incidence decline 17.2% per year**	Reductions in serogroup C IMD among program eligible and ineligible age groups suggest both direct and indirect MCCV vaccine program impact
Kinlin, 2009	**Canada**, Ontario	MCCV	Routine	12 months and catch up for children aged 12 or 15–19 year-olds	All ages	Invasive meningococal disease	Decline from 5.48 to 4.26 cases per 1 000 000. Rate of serogroup C decreased 50.12% while non-C decreased by 5.61%.	**Rate of serogroup C decreased 50.12%**	Conjugate serogroup C vaccination in Ontario appears to have had a direct effect on IMD incidence in children and adolescents, who are at greatest risk of invasive infection. The downward trend observed in older, unvaccinated age groups suggests that there are also herd benefits to immunization
De Wals, 2004	**Canada**, Quebec	MCCV	Campaign	One dose to all people aged 2 months to 20 years	All ages	Serogroup C meningococcal disease	The incidence rates were 1.04 per million in 1996–2000, 7.84 in 2001 (P .001), and 3.63 in 2002. For the age group targeted for vaccination, the incidence increased from 2.90 per million in 1996–2000 to 21.47 in 2001, then decreased to 3.26 in 2002. For those 21 years and older, the incidence was similar between 2001 (3.26) and 2002 (3.77)	**VE 96.8%**	The new conjugate vaccine was effective in controlling an emerging epidemic of serogroup C meningococcal disease, as well as providing short-term protection across a wide age range
O Maoldomhnaigh 2016	**Ireland**	MCCV	Routine	Under 19 years of age	Under 19 years of age	Menigococcal cases	From 14.75 per 100 000 (1999) to 2 per 100 000 (2011), and 98% of disease caused by serogroup B and a national CFR of 3.6%	**Not expressed**	Despite the meningococcal C vaccination campaign, invasive meningococcal disease continues to cause serious morbidity and claim lives. Group B infections remain dominant. As children who die often present with fulminant disease, preventive strategies including use of meningococcal B vaccine are needed to avert death and sequelae
De Waure, 2015	**Italy**	MCCV	Routine + catch-up	13–15m; catch-up at 11–18yrs	<1 year; 1–4 yrs; 5–9 yrs; 10–14 yrs; 15–24 yrs; 25–64 yrs; 65 yrs and over	“Cases are defined according to clinical and laboratory criteria. A confirmed case is defined as a patient with a compatible clinical illness and a laboratory confirmation such as the detection -through microscopic direct examination, culture or polymerase chain reactionof N. meningitidis from a normally sterile site or the identification of the polysaccharide antigen in the cerebrospinal fluid.”	Incidence pre-intro (1994–2005) for all cases: 0.40/100 000. Incidence post-intro (2006–2012) for all cases: 0.28/100 000. Incidence pre-intro (1994–2005) for MenC cases: 0.07/100 000. Incidence post-intro (2006–2012) for MenC cases: 0.05/100 000. Incidence pre-intro (1994–2005) for Untyped cases: 0.20/100 000. Incidence post-intro (2006–2012) for Untyped cases: 0.07/100 000	**Not expressed**	Our results suggest that MCC had an impact in decreasing the incidence of N. meningitidis C related IMD. However, data on typing are incomplete and efforts are needed to make them available for studying the need and the impact of other meningococcal disease
Pascucci, 2014	**Italy**, Emilia Romagna	MCCV	2003 – at risk; 2006 – routine	12–15 months and 14–15 years	<1yr; 1–4 yrs; 5–14 yrs; 15–24 yrs; 25–64 yrs; 65 and over; all ages	Presence of N. meningitidis (either detected by standard culture or by DNA amplification of a pathogen specific genomic target) in a cerebrospinal fluid (CSF) sample with hospitalization	The average incidence of meningitis caused by meningococcus declined from 0.54/100 000 (years 2000–2005) to 0.33/100 000 (years 2006–2012). The total number of notified serogroup C cases dropped 0.2/100 000 in 2000–2005 to 0.06/100 000 (–70.1%) in 2006–2012. This change reached –100% (no case notified) and -83.1% in the two target age groups 1–4 and 15–24	**Incidence decline for serogroup C was 70.1%. VE was estimated as 90% or above in the age groups targeted by the vaccination campaign**	No case of serogroup C related infection was observed since 2006 in children aged 1–4 years. These findings suggest that the single-dose vaccination strategy against serogroup C N. meningitidis targeted to the age groups 12–15 months and 14–15 years was effective in the Emilia-Romagna population. However, the occurrence of two cases of meningitis in a 5-month child and in a 9-years child suggests caution and careful consideration in surveillance for the next years
Bechini, 2012	**Italy**, Tuscany	MCCV	Routine	3 doses at 3, 5, and 13 months of age and catch up until age 6 with a single dose	Incidence rates were calculated for the following age groups: <1 year; 1–4 years; 5–14 years; 15–20 years; 21–30 years;31–49 years; 50–64 years;≥65 years.	invasive meningococcal C disease	The highest incidence rates were observed in infants, ranging from 3.2/100 000 in 2005 to 9.3/100 000 in 2010. Incidence rates of meningitis decreased from 2005 to 2010 in the other age groups: an incidence rate of 5.1/100 000 was registered in subjects of 1–4 years in 2005. Decrease in the incidence rate was also observed in subjects aged 5–14 years	**Not expressed**	Progressively increasing vaccination coverage
Mooney 2004	**Scotland**	MCCV	Routine	All people under 20 years of age	All ages	serogroup C meningo disease	From 15.8 incidents per 100 000 subjects in 1999 (95% confidence interval [CI], 11.3–20.3) to 0.7 incidents per 100 000 subjects in 2001 (95% CI, 0.3 to 1.6), for subjects <5 years old, and from 6.7 incidents per 100 000 subjects in 1999 (95% CI, 5.1–8.3) to 1.5 incidents per 100 000 subjects in 2001 (95% CI, 0.7–2.3), for subjects 5–19 years old	**VE 99.23%**	The MCC vaccine program has been highly effective in Scotland, leading to substantial reductions in serogroup C meningococcal disease and meningococcal mortality, with no adverse effects on other groups
Cano, 2004	**Spain**	MCCV	Routine	Infants (not further specified)	All Age groups	Lab confirmed meningococcal disease	Relative risk of suffering meningococcal diseaes was 0.58 (1999–2000) Before / (2002–2003) After Relative risk of suffering meningococcal C diseaes was 0.42 (1999–2000) Before / (2002–2003) After Relative risk of suffering meningococcal B diseaes was 0.75 (1999–2000) Before / (2002–2003) After Relative risk of suffering meningococcal diseaes no groupable was 1.59 (1999–2000) Before / (2002–2003) After It has been estimated that the risk of contracting the disease of this serogroup fell by 58% if we compare the incidence of the last epidemiological year in the study with that of the season before the conjugate vaccine was introduced.	**RR 0.42**	The incidence of meningococcal disease, especially serogroup C, has fallen sharply during the last three epidemiological seasons in Spain covered by this study
Garrido, 2014	**Spain**	MCCV	Routine	2, 4, 6 months of age (in 2006, a boster dose during the second year was added)	All ages	Lab confirmed meningococcal disease	Between 1997/98 and 1999/00 seasons the MenC incidence rates remained around 0.9 cases per 100 000 pop. Between 2000/01 and 2005/06 seasons the MenC incidence rates remained around 0.37 cases per 100 000 pop. Between 2006/07and 2012/13 seasons the MenC incidence rates remained around 0.14 cases per 100 000 pop. Routine-2 schedule displayed higher VE than routine-1 schedule (99.3% vs. 90.2%, p < 0.001). VE ≤1 year since vaccination for routine-1 (97.5%) and routine-2 (99.8%) was slightly different. However, >1 year since vaccination loss of VEwas higher for routine-1 (81.5% vs. 89.1%, p < 0.001).	**VE 94.83%**	The meningococcal C conjugate vaccination programme has been extremely successful in controlling the disease and continues to be evaluated and adapted to the changes in the epidemiology of the disease to ensure long-term vaccine protection
Larrauri, 2005	**Spain**	MCCV	Routine	2,4,6 months and catch up (variable ages)	Children under 6 years of age	Serogroup C meningococcal disease	Among such children, 42 cases were reported in the last epidemiological year analysed versus 268 cases in the season preceding vaccination, a reduction of 85% in incidence in this age group. Catch-up 7 months to 5 years effectiveness 97.8 (96.0–98.8) Routine 2, 4, and 6 months 98.4 (95.7–99.4)	**incidence decline 85%. 97.8% VE for children vaccinated in the catch-up campaign and 95.2% for those who had been routinely vaccinated in infancy**	The vaccine registered high short-term VE values but there has been some loss of VE with time. Four years after vaccination, vaccine protection levels exceeded 94% in cohorts immunised during the campaign. Among children vaccinated in routine childhood immunisation programmes, however, long-term VE loss was greater.
Salleras 2003 (Impact...)	**Spain**	MCCV	Routine and campaign	Routine: 2, 4, 6 months of age. Campaign: children aged between 2 months and 6 years	Children under 6 years of age	Lab confirmed meningococcal disease	Reported cases of serogroup C meningococcal disease decreased sharply after the mass vaccination campaign carried out during the year 2000 (18 cases in 2001 compared with 46 in 2000 observed in the <6 years age group (only 2 cases in 2001, both in non-vaccinated children, compared with 27 cases in 2000).Vaccination effectiveness in children <6 years was 100% (94.27–100%).	**Not expressed**	The results of this study show the high level of effectiveness of the meningococcal C conjugated vaccine in the short term
Cruz Rojo 2005	**Spain**, Andalucia	MCCV	Campaign	Under 10 years of age	<10 years	Meningococal disease	The annual incidence in < 10 years pre vaccination 8.2 per and post vaccination 2.0 per 100,000 inhabitants, this difference being Statistically significant. Conversely, In the population over 10 years old has produced neither that downward trend In the incidence, nor a significant difference between the average annual rates of both periods (rates of 1.2 and 1.0 respectively)	**Not expressed**	The absence of vaccine failure and the impact observed on the incidence of serogroup C meningococcal disease in children under 10 suggests the effectiveness of this new conjugate vaccine
Salleras, 2003 (Dramatic...)	**Spain**, Catalonia	MCCV	Routine (2000) and campaign (2001, 2002)	Routine: 2, 4 ,6 months. Campaign: 6–19 year-olds	All ages	Lab confirmed meningococcal disease	The accumulated 4-weekly incidence rates in children <6 years of age show the dramatic decline in disease incidence during the 2000–2001 epidemic season a decline which was maintained during the 2001–2002 epidemic season. In contrast, in the 6–19 years age group, the moderate decrease observed during the 2000–2001 epidemic season was not maintained during the 2001–2002 epidemic season. Vaccination effectiveness in children <6 years was 100% (94.27–100%)	**VE 100%**	The available data seem to confirm that there has been no substitution of serogroup C by serogroup B after mass vaccination with the conjugated vaccine, at least in the short term. However, this subject still preoccupies expert opinion and only the future evolution of the disease will provide a definitive answer
Romero 2011	**Spain**, Galicia	MCCV	Routine infant; Catch-up	2,4,6 months; one time catch-up all children up to 19yrs; another one time catch-up 13–25 years who had not received the conjugate vaccine	13–25 yrs	Isolation of Neisseria meningitidis in a totally sterile environment, or detection of Neisseria meningitidis genome in a sterile environment or detection of Neisseria meningitidis in CSF or diplococcus gram-negative display in CSF	Incidence in 2004–2005 was 0.84 cases per 100 000 – reduced to 0.18 cases per 100 000 in 2007–2008	**Not expressed**	In Galicia, a series of vaccination campaigns, particularly focusing on high-risk groups, has shown high effectiveness, with a marked reduction in the disease incidence in the vaccination cohort accompanied by a relevant reduction in the overall population.
Morales, 2016	**Spain**, Navarra	MCCV	Routine + catch-up	Routine: newborns/infants; catch-up campaigns: in 2000 – <6 yrs and in 2004 <17 yrs	<15 yrs and adults (all ages above 15yrs)	Presence of N. meningitidis via PCR, isolation from CSF, identification of N. meningitidis from a sterile site	Between 1995 and 1999, the mean annual incidence of meningococcus C disease was 20.81 and 14.13 cases per 100 000 in children younger than 1 year and 1 to 4 years, respectively. The first part of the post-vaccination period (2001–2003), the incidence was reduced by 100% in the 2 groups less than 5 years old (p <0.001). In the second part of the post-vaccination period (2004–2014), declines in the incidence of 95% were consolidated in the 1-year-olds (p <0.001) and 100% in the 1-to-4-year-olds ( P <0.001)	**Incidence decline 100%and 95%. VE 96%**	The MenCC vaccination program has been succesful in decreasing the incidence rate of serogroup C meningococcal disease in Navarra, and schedule changes have maintained high vaccine effectiveness throughout the study period
Parikh, 2016	**UK**	4CMenB	Routine	2, 4 months of age with oportunistic catch-up for 3 and 4-months old	Infants born on or after May 2015	Lab confirmed meningococcal disease	Two-dose vaccine effectiveness was 82·9% (95% CI 24·1–95·2) against all MenB cases	**50% incidence rate ratio (IRR) reduction. VE 82.9%**	The two-dose 4CMenB priming schedule was highly eff ective in preventing MenB disease in infants. Cases in vaccine-eligible infants halved in the first 10 months of the programme
Martin, 2014	**UK**	MCCV	Routine + catch-up	“Infant”; catch-up 19 yrs	<15 yr	“We defined diseases according to International Classifi cation of Disease (ICD) codes (table 1) and included cases if the diagnosis was recorded as the primary or one of the secondary diagnoses.”	Person-based admission rates per 100 000 children decreased by 66%, from 26·68 (25·59–27·77) in 1999 to 9·10 (8·48–9·71) in 2011	**Admission rates decreased by 66%**	Vaccine-preventableinvasive bacterial disease in children has decreased substantially in England in the past five decades, most notably with the advent of effective conjugate vaccines since the 1990s. Ongoing disease surveillance and continued development and implementation of vaccines against additional pneumococcal serotypes and serogroup B meningococcal disease are important
Trotter, 2004	**UK**	MCCV	Routine	2,3,4 months and catch-up to children younger than 18 years of age	All ages	Laboratory-confirmed meningococcal serogroup C disease	Vaccine effectiveness was high (83%) in all children who had received MCC vaccines in the catch-up campaign at age 5 months to 18 years. Overall, routine infant vaccination was estimated to be 66% effective, but clear differences were noted according to time since vaccination	**VE 66% to 83%**	Vaccine effectiveness remained high in children vaccinated in the catch-up campaign (aged 5 months to 18 years). However, for children vaccinated in the routine infant mmunisation programme, the effectiveness of the MCC vaccine fell to low levels after only 1 year. The number of individuals in these cohorts remains low, but alternative routine immunisation schedules should be considered to ensure high levels of protection are sustained

**TABLE 2c.** 

First Author, year	Country and state	Type of vaccine	Serogroups included in the vaccine	Specific vaccine	Study type	Data source	Study period	Vaccine introduction or campaign date
Kupek, 2001	**Brazil**, Santa Catarina	Polysaccharide	C	MenC polysaccharide	Impact and effectiveness	National Surveillance system	March 1995 to March 1997	1 March 1996
De Wals, 2001	**Canada**, Quebec	Polysaccharide	A, C, Y, W	ACYW and A+C	Impact and effectiveness	Cases reported to regional health authorities by clinicians, hospital laboratories, and the provincial laboratory serving as a reference centre for N. meningitidis	1990–1998	Dec 1992 to March 1993
Perez-Rodriguez, 1999	**Cuba**	Polysaccharide vaccine	B+C	VA-Meningo-BC	Impact	Direct Information System	1991–1996	1991
Goicoechea Saez, 2003	**Spain**, Valencia	Polysaccharide vaccine	A+C	Menpovax A+ C (ploysaccharide)	Impact and effectiveness	Clinical records from public hospitals	1996–2000	Sep to Dec 1997
Pereiro, 2001	**Spain**, Valencia	Polysaccharide	A+C	A+C (two different vaccines were used)	Impact	Population-based active surveillance	1996–1998	Oct to Dec 1997
Bergman, 2000	**UK**	Polysaccharide	A+C	Menpovax A+ C (ploysaccharide)	Impact	Hospital inpatient summaries	1989–1997	Late 1992

**TABLE 2d.** 

First Author, year	Country and state	Specific vaccine	Vaccination STRATEGY (Routine or campaign?)	Vaccination schedule used	Age groups on which impact was measured	Case definition	Effectiveness or impact as reported	Impact estimation	Conclusion of the study (short)
Kupek, 2001	**Brazil**, Santa Catarina	MenC polysaccharide	Campaign	All children between 6 months and 14 years of age	All ages	Lab confirmed meningococcal disease	In general population of Sta Catarina, VE was 74.3% (52.7% to 99.6%), and in children 6 months to 14 years, VE was 93.1% (85.2% to 100%)	VE 74.3% and for children 6 months to 14 years of age 93.1%	Group C menigococcal vaccine is effective in reducing the occurrence of meningococcal disease in children 6 months to 14 years of age, and that the ration of rate rations (RRR) is a useful method to evaluate vaccine effectiveness
De Wals, 2001	**Canada**, Quebec	ACYW and A+C	Campaign	All persons between 6 months and 20 years of age	All ages	Meningococal disease between 1990–1998 and culture-proven serogroup C meningococcal disease between 1993–1998	The incidence of serogroup C disease decreased after the mass immunization campaign, from 1.4 per 100 000 in 1990–1992 to 0.3 per 100 000 in 1993–1998, and the overall incidence of other serogroups remained stable at 0.7 per 100 000, with a small increase in the proportion of cases caused by serogroup Y Protection from serogroup C MCD was indicated in the first 2 years after vaccine administration (VE, 65%; 95% confidence interval [CI], 20%-84%), but not in the next 3 years	VE, 65% first 2 years, afterwards 0%	Serogroups C polysaccharide vaccine is affective for controlling outbreaks in teenaged individuals but should not be used in children younger than 2 years. The mass campaigndid not induce significant serogroup switching
Perez-Rodriguez, 1999	**Cuba**	VA-Meningo-BC	Routine	3 and a half months, 5 and a half months	Children 1 to 4 years of age	Menigococcal cases with lab confirmation	Incidence density in 1991: 10.8 per 100 000. Incidence density in 1995: 0.67 per 100 000. Incidence density in 1996: 0.68 per 100 000.	Not expressed	The changes observed in the incidence of meningococcal disease and the displacement of higher risk to older age groups of 3 and 4 years of age constitute an important support to continue the administration of the vaccine in the routine program, even more so if a booster dose is added to the schedule
Goicoechea Saez, 2003	**Spain**, Valencia	Menpovax A+ C (ploysaccharide)	Campaign	18 months to 19 years of age	Children under 15 years of age	Invasive meningococal disease	The rate of incidence by serogroup C in children under age 15 dropped following the vaccination campaign from 5.82/105 habitants in 1997 to 1.68/105 habitants in 1998. Rates similar to those prior to the time prior to the vaccination recorded three years subsequent to the campaign, showing an increase in the disease caused by serogroup B over the last 2 years. Sixty-one percent of the sequelae were among children under 5 years of age. Lethality was higher for serogroup C. Vaccination efficacy three years subsequent to the campaign was 83.7% for the 5–14 age range and 69.1% for the 19month-4 year age range	VE 83.7% for 5–14 years and 69.1% for 19 m-4 years	The polysaccharide vaccine was shown to be effective for halting the outbreak. The drop in the incidence of serogroup C can be attributed to the vaccination efficacy achieved
Pereiro, 2001	**Spain**, Valencia	A+C (two different vaccines were used)	Campaign	Persons between 18 months and 19 years of age living in the region	Children under 15 years of age	Meningococcal disease lab confirmed or clinical manifestations	The cumulative incidence increased from 10.6: 100 000 in 1996 to 15.2:100 000 in 1997, but decreased to 7.9:100 000 in 1998, primarily due to a reduction in the incidence of serogroup C disease	Not expressed	Meningococcal polysaccharide vaccine seems to be an effective public health tool for the management of this serious communicable disease
Bergman, 2000	UK	Menpovax A+ C (ploysaccharide)	Routine	All recruits	1 dose of vaccine to all recruits	Bacterial meningitis or meningococcemia	The crude relative risk for meningococcal infection in recruits after vaccination compared with the period before vaccination was 0.94 (95% confidence interval (CI) 0.40 – 2.22) and the corresponding relative risk for trained personnel was 0.20 (95% CI 0.09 – 0.44)	RR 0.94	From 1993 onwards, no further clusters of group C infection were reported and incidence of meningococcal disease among trained soldiers fell, but there was no significant reduction in the overall incidence of meningococcal disease in recruits

Only four of the studies selected for this review originated in Latin America and the Caribbean (LAC). This was not unexpected since only three countries in the area have introduced a meningococcal vaccine into their routine schedule and conducting these types of studies in LAC is challenging due to the area’s insufficient surveillance of meningococcal disease. All four studies obtained their disease-burden data from Regional public health laboratories. This reinforces the premise that regional surveillance systems are of paramount importance to estimating the impact of vaccine introduction, and therefore, should be adequately maintained and strengthened (13).

## CONCLUSIONS

These 32 studies have suggested or shown a reduction in cases and deaths due to vaccine-serotype invasive meningococcal disease after introduction of a meningococcal vaccine to the routine schedule or as part of a mass campaign. When considering the meningococcal vaccine, each LAC country should take into account its particular epidemiologic profile, as well as the cost of these vaccines, only one of which—the conjugate tetravalent ACYW-135— is currently offered through the PAHO Revolving Fund (US$ 20.30 per dose; 47).

LAC countries should also strengthen their epidemiological surveillance systems to include serogroup-specific surveillance of invasive meningococcal disease. Adequate surveillance can measure the magnitude of this public health issue, thereby informing decision makers, while setting an epidemiologic foundation for conducting future vaccine impact and effectiveness studies.

### Disclaimer.

Authors hold sole responsibility for the views expressed in the manuscript, which may not necessarily reflect the opinion or policy of the *RPSP/PAJPH* and/or PAHO.

## Footnotes

An evidence-based minimum set of items for reporting in systematic reviews and meta-analyses, focusing on the reporting of reviews evaluating randomized trials, but also used as a basis for reporting systematic reviews of other types of research, particularly evaluations of interventions. For more information visit: https://www.prisma-statement.org/
